# Dissociative Tremor Response with Pallidal Deep Brain Stimulation in Parkinson’s Disease

**DOI:** 10.5334/tohm.568

**Published:** 2020-12-16

**Authors:** Anson Wang, Eric Molho, Yingmai Yang, Julie Pilitsis, Adolfo Ramirez-Zamora

**Affiliations:** 1 Department of Neurology, University of Florida, Gainesville, FL, US; 2 Department of Neurology, Albany Medical Center, Albany, NY, US; 3 Peking Union Medical College Hospital, Beijing, CN; 4 Department of Neurosurgery, Albany Medical Center, Albany, NY, US; 5 Department of Neuroscience & Experimental Therapeutics, Albany Medical College, Albany, NY, US

**Keywords:** Deep brain stimulation, GPi, VIM, Tremor, Parkinson’s disease

## Abstract

**Background:**

Pallidal and subthalamic targets are commonly used for deep brain stimulation in Parkinson’s disease (PD), with similar efficacy for resting tremor control. However, neuromodulatory effects on kinetic and postural tremor in PD is less clear.

**Case Report:**

We present a 67-year-old PD patient with marked dissociative tremor response following pallidal neuromodulation. We observed excellent resting tremor suppression, but postural and kinetic tremors remained severe, requiring additional thalamic VIM stimulation for management.

**Discussion:**

Our findings illustrate the phenotypical differences in PD and differential response to diverse tremor characteristics with distinctive stimulation targets. Additional studies are necessary to understand these differences.

## Introduction

High frequency deep brain stimulation (DBS) of the subthalamic nucleus (STN) and globus pallidus interna (GPi) are safe and effective treatments for alleviating motor symptoms in Parkinson’s disease (PD) [1,2]. While levodopa is the main medication used for management of PD, over time, patients develop motor complications, fluctuations with reduced and unpredictable medication responses, dyskinesia, and neuropsychiatric complications [3]. DBS may potentially reduce the usage of dopaminergic medications and has been highly effective for management of tremor (responsive or unresponsive to levodopa).

Results of randomized clinical trials in PD and a recent meta-analysis revealed comparable efficacy between STN and GPi targets in tremor suppression and achieving motor benefits [4,5,6,7]. The ventral intermediate nucleus (VIM) of the thalamus has traditionally been considered as the main target for tremor control. Several reports indicate marked tremor benefit with thalamic VIM DBS in parkinsonian tremor with persistent efficacy over 5 years [8,9,10]. However, other motor features such as akinesia, rigidity, and postural instability remain unresolved, without clear improvement in Unified Parkinson’s Disease Rating Scale (UPDRS) motor scores. Due to methodological difficulties, the effects of DBS on different tremor components in PD have not been clearly established and most studies relied on singular tremor scores from the UPDRS for assessment. In this report, we present a case of PD who was treated with high frequency GPi DBS followed by VIM thalamic stimulation due to limited postural and kinetic tremor response with pallidal neuromodulation despite resolution of resting tremor.

## Clinical Presentation

Patient is a 67-year-old man diagnosed with PD, presenting with debilitating resting, kinetic, and postural tremors for several years. Patient noted a unilateral, right hand, low frequency resting tremor, followed by postural and kinetic tremors in the same hand over the following months. Tremor remained unilateral and highly asymmetric for a couple of years. Subsequent tremor progression to his left hand and increasing frequency and amplitude of postural and kinetic tremors eventually lead to marked functional difficulties. He developed bradykinesia, dysarthria, hypokinesia, and a prominent resting tremor while walking or when distracted. Levodopa (titrated up to 700 mg/d in divided doses over time) improved parkinsonism, except for all tremor components. There were no reports of dyskinesia or motor fluctuations. His UPDRS-motor score in the OFF-medication state was 16 primarily due to tremor.

Tremor was unresponsive to levodopa despite reduction in bradykinesia, rigidity and abnormal posture. A DaT scan showed asymmetrical dopaminergic deficit with the left striatum more severely affected. DBS was considered due to marked functional difficulties related to tremor. He had a severe, persistent resting tremor with moderate amplitude, in addition to postural and kinetic tremors in both hands—although to a greater degree on the left hand. His pre-operative total UPDRS score was 38, scoring 6/16 in part I, 16 points on part II (activities of daily living) and 16 points on section III (motor scores) in the off-medication state (Video 1). During multidisciplinary pre-surgical evaluation, the patient had moderate cognitive impairment and reported sustained depression (spanning 1 week or longer), impaired swallowing and speech, and illegible writing.

**Video 1:** **Clinical assessments of motor features.** Clinical assessments of motor features. Evaluations were conducted prior to neurostimulation, 6 months after left GPi DBS, and 3 months post-left VIM DBS under maximized programming in the ON DBS state. Following pallidal DBS, the patient exhibited excellent control over resting tremor, although he had severe, residual postural and kinetic tremors which lead to marked functional disability. Persistent tremors resolved following thalamic VIM DBS.

**Figure 1:**
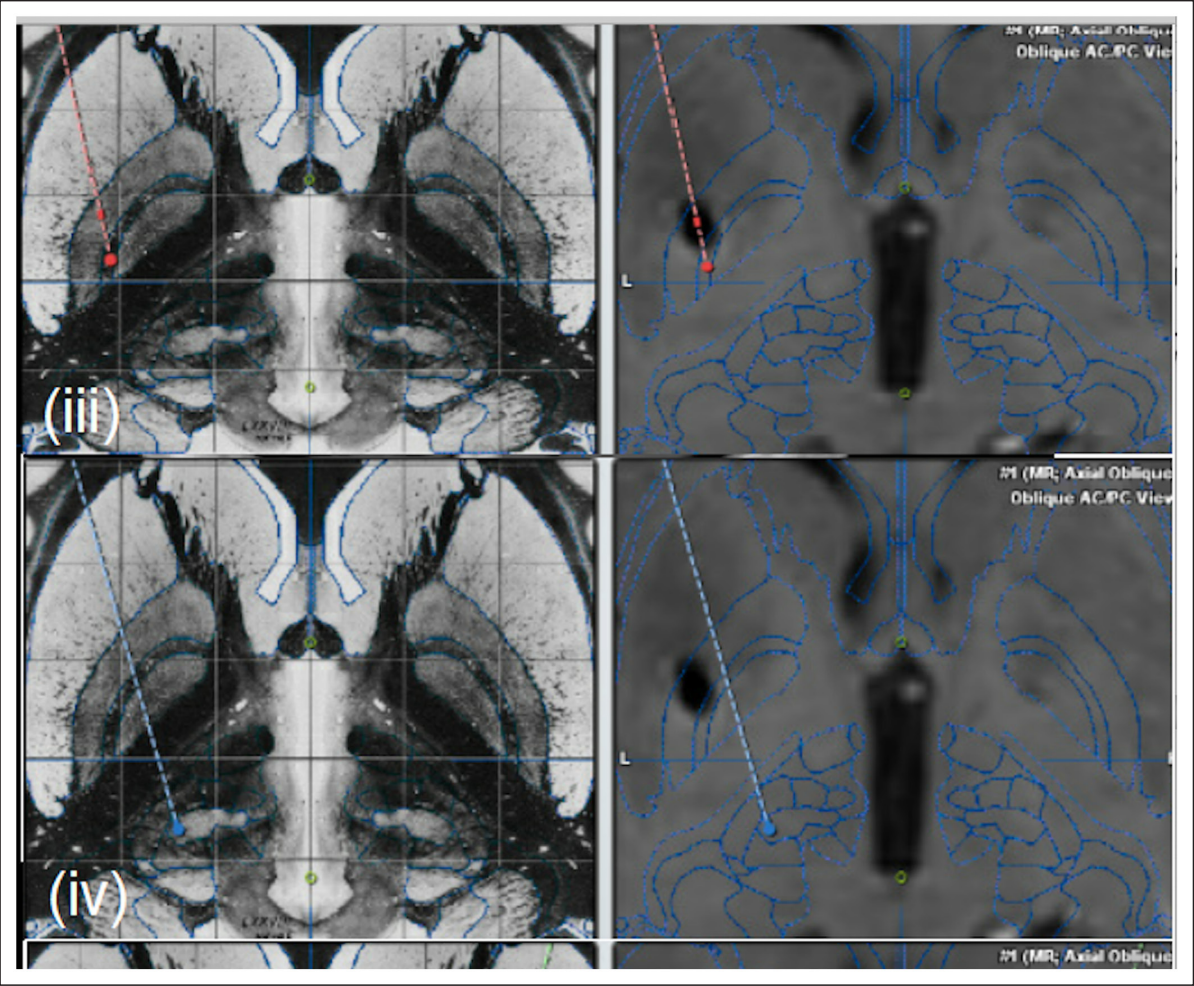
Localization of DBS electrodes. Electrodes locations were confirmed using BrainLab Stereotactic planning module. Following fusion of pre-operative MRI and post-operative CT, the electrodes were marked on the CT images. Atlas views were matched to the MRI images based on anatomy. **(iii)** Matched atlas view of the Lt. GPi lead. **(iv)** Matched atlas view of the Lt VIM lead.

Concerns regarding cognitive function and axial symptoms led to the selection of staged bilateral DBS, starting with the left GPi. Six months following lead placement, the patient noted excellent control on resting tremor. However, he had minimal improvement in postural and kinetic tremors. We implemented a variety of programming configurations for persistent tremors. His initial monopolar review showed adequate clinical thresholds with all electrodes ranging from 2.5 to 4.0 V (PW of 90 µs and frequency of 130 Hz). We first increased amplitude in most ventral contacts and assessed for tremor control; this was followed by a double unipolar configuration at higher voltages. Programming changes were monitor for at least one or two weeks before additional adjustments. Due to the lack of benefit with most ventral contacts, we activated dorsal contacts independently following a similar sequence and then increased PW up to 150 µs. This was further followed by adjustments to higher stimulation frequencies (180 Hz). Side effects included spastic dysarthria and facial muscle contractions with higher amplitudes due to activation of corticospinal tracts. Unfortunately, postural and kinetic tremor remained severe. (Video 1). To address these deficits, we decided to add a left thalamic VIM lead. Three months after the ipsilateral (left) VIM DBS, he noted resolution of kinetic and postural tremors. Post-operative neuroimaging showed optimal positioning of both DBS leads (Figure 1). Adverse effects related to DBS programming included moderate dysarthria. Final programming settings are as follows to minimize side effects such as moderate to severe dysarthria (mixed type) resulting from activation of both leads; left GPi lead: bipolar (3+, 2–), 3.5V, 90µs, 180Hz and left VIM lead: bipolar (8–, 9+), 3.6V, 90µs, 180Hz.

## Discussion

As a cardinal motor feature in PD, tremor is present in more than 80% of patients and can be unresponsive to levodopa [11]. Tremor distribution diverges among PD patients with variable resting, postural, and kinetic tremors. A retrospective study of 332 patients with idiopathic PD found that 67.5% (224/332) of patients presented with a resting tremor, 40.0% with a kinetic tremor, and 30.1% with a postural tremor [12]. In this cohort, 37.0% were diagnosed with two or more tremor types and 26.5% with no tremor [12].

Therapeutic interventions such as DBS suppress tremor in PD and improve patient’s quality of life [1,2]. Early studies demonstrated marked benefit in Parkinson’s disease tremors with thalamic VIM DBS—but limited benefits on alleviating rigidity and bradykinesia [13,14]. While standard stimulation targets such as the STN and GPi are effective in managing tremor, rigidity, bradykinesia, and dyskinesia, many studies suggest no significant differences in their efficacy [4,5,6,7]. Cognitive concerns dictated initial pursuit of pallidal DBS as we anticipated adequate tremor control as described.

In certain circumstances, PD patients might benefit from thalamic stimulation when presenting with an extremely debilitating tremor profile [7]. Thalamic VIM DBS is traditionally used as the target of choice for essential tremor [14], reducing tremor in 85–95% of patients. Thalamic DBS is also recognized for its long-term efficacy in tremor suppression [15,16].

This case study illustrates the potential for disparity in response to pallidal and thalamic DBS with different tremor phenotypes in PD. In this case, stimulation of the GPi was associated with a complete resolution of the resting tremor, but persistent postural and kinetic residual tremors required additional DBS surgery targeting the VIM. The mechanisms responsible for the different tremor types in PD are not well understood and very few studies have characterized the efficacy of different DBS targets on specific tremor phenotypes. Among these, a blinded study by Hubble et al. found that thalamic stimulation in PD patients resulted in significant improvements in Clinical Tremor Rating Scale scores for resting, kinetic, and distal/proximal postural tremors (score changes of 3.20, 1.60, 2.50, and 2.20 from baseline respectively; p < 0.01) [17]. Most clinical studies to date do not incorporate tremor rating scales as a primary motor outcome, leading to incomplete and limited assessments of postural and kinetic tremors with the UPDRS [18,19].

Helmich and colleagues (2012) proposed the dimmer switch model to explain the causes and effects of the parkinsonian resting tremor. They proposed a theory which states that the basal ganglia triggers the cerebello-thalamo-cortical network to produce tremor-related responses at the motor cortex (where the two circuits converge) [20]. Thus, it appears plausible that contributions from the striato-pallidal circuit cause the tremor on/offset (analogous to a light switch) and contributions from the cerebello-thalamo-cortical system modulate the intensity of the tremor (analogous to a light dimmer). In levodopa refractory PD individuals such as the patient discussed herein, both networks are likely implicated. The dissociative resting tremor response after pallidal stimulation may be due to disruptions in the pathological tremor initiation process while the limbs are “en repose”. Involvement of the cerebello-thalamo-cortical system is critical in modulating active movements and their amplitudes, which would require disruption of pathological oscillations in this network (with thalamic DBS) to achieve tremor suppression in our patient. Therefore, in certain PD patients, adequate tremor control may require modulation of both networks.

While this case study supports the application of using additional DBS targets to manage resistant or residual tremor after initial DBS, further research and comparative studies are necessary. Future studies should focus on elucidating the effectiveness of standard DBS targets in PD-specific tremor subtypes, collecting details and objective tremor assessments, and investigating the potential functional and structural connective differences among targets.
